# Impact of gut microbiome on dyslipidemia in japanese adults: Assessment of the Shika-machi super preventive health examination results for causal inference

**DOI:** 10.3389/fcimb.2022.908997

**Published:** 2022-09-02

**Authors:** Yuna Miyajima, Shigehiro Karashima, Kazuhiro Ogai, Kouki Taniguchi, Kohei Ogura, Masaki Kawakami, Hidetaka Nambo, Mitsuhiro Kometani, Daisuke Aono, Masashi Demura, Takashi Yoneda, Hiromasa Tsujiguchi, Akinori Hara, Hiroyuki Nakamura, Shigefumi Okamoto

**Affiliations:** ^1^ Department of Clinical Laboratory Science, Faculty of Health Sciences, Institute of Medical, Pharmaceutical and Health Sciences, Kanazawa University, Kanazawa, Japan; ^2^ Institute of Liberal Arts and Science, Kanazawa University, Kanazawa, Japan; ^3^ AI Hospital/Macro Signal Dynamics Research and Development Center, Institute of Medical, Pharmaceutical and Health Sciences, Kanazawa University, Kanazawa, Japan; ^4^ Advanced Health Care Science Research Unit, Institute for Frontier Science Initiative, Kanazawa University, Kanazawa, Japan; ^5^ School of Electrical, Information and Communication Engineering, College of Science and Engineering, Kanazawa University, Kanazawa, Japan; ^6^ Department of Endocrinology and Metabolism, Kanazawa University Hospital, Kanazawa, Japan; ^7^ Department of Hygiene, Graduate School of Medical Sciences, Kanazawa University, Kanazawa, Japan; ^8^ Department of Health Promotion and Medicine of the Future, Kanazawa University, Kanazawa, Japan; ^9^ Faculty of Transdisciplinary Sciences, Institute of Transdisciplinary Sciences, Kanazawa University, Kanazawa, Japan; ^10^ Department of Public Health, Graduate School of Advanced Preventive Medical Sciences, Kanazawa University, Kanazawa, Japan

**Keywords:** gut microbiome, sex, dyslipidemia, causal inference, linear non-gaussian acyclic model

## Abstract

Dyslipidemia (DL) is one of the most common lifestyle-related diseases. There are few reports showing the causal relationship between gut microbiota (GM) and DL. In the present study, we used a linear non-Gaussian acyclic model (LiNGAM) to evaluate the causal relationship between GM and DL. A total of 79 men and 82 women aged 40 years or older living in Shika-machi, Ishikawa Prefecture, Japan were included in the analysis, and their clinical information was investigated. DNA extracted from the GM was processed to sequence the 16S rRNA gene using next-generation sequencing. Participants were divided into four groups based on sex and lipid profile information. The results of one-way analysis of covariance, linear discriminant analysis effect size, and least absolute value reduction and selection operator logistic regression model indicated that several bacteria between men and women may be associated with DL. The LiNGAM showed a presumed causal relationship between different bacteria and lipid profiles in men and women. In men, *Prevotella 9* and *Bacteroides* were shown to be potentially associated with changes in low- and high-density lipoprotein cholesterol levels. In women, the LiNGAM results showed two bacteria, *Akkermansia* and *Escherichia/Shigella*, had a presumptive causal relationship with lipid profiles. These results may provide a new sex-based strategy to reduce the risk of developing DL and to treat DL through the regulation of the intestinal environment using specific GM.

## Introduction

Dyslipidemia (DL) is a major risk factor for the development of cardiovascular disease (CVD), which is one of the leading causes of death worldwide. Therefore, aggressive diagnosis and treatment of DL is important to reduce the incidence of CVD and its associated mortality (Bartlomiejczyk et al., 2019). In most cases, DL is closely related to lifestyle practices such as excessive energy intake, alcohol consumption, and smoking, which in turn adversely impact the blood levels of low-density lipoprotein cholesterol (LDL-C), high-density lipoprotein cholesterol (HDL-C), and triglycerides (TG) (Eberle et al., 1991).

In recent years, the gut microbiota (GM) has received much attention with regard to disease pathogenesis (Shreiner et al., 2015; Kho and Lal, 2018). Its composition has been shown to vary greatly with lifestyle, age, and sex (Yatsunenko et al., 2012; Takagi et al., 2019). In addition, GM play an important role in the regulation of many metabolic processes in the host, including energy homeostasis, glucose regulation, and lipid metabolism (Le Chatelier et al., 2013; Sanna et al., 2019; Visconti et al., 2019). Several studies have shown that GM regulate lipid levels in response to diet and host lipid metabolism. Animal studies have shown that a high-fat diet alters the GM of the host (Bäckhed et al., 2007; Devkota et al., 2012). Fu et al. reported the association of GM with TG and HDL-C levels in a cohort study (Fu et al., 2015). Takagi et al. found that several intestinal bacteria, including *Escherichia*, were associated with DL in Japanese participants (Takagi et al., 2020).

One of the causal inference methods that has been proposed for the assessment of the causal structure of variables, is the linear non-Gaussian acyclic model (LiNGAM) (Shimizu et al., 2011). An important aspect of the LiNGAM is that it can identify more generative structures when the data are non-Gaussian than when they are in a traditional Gaussian setting. Variables such as GM are known to be non-Gaussian, which makes this method suitable for the present analysis.

Most reports that have examined the causal relationship between GM and DL indices have been based on animal experiments, and epidemiological studies of GM in humans have only examined the association, not causation. Therefore, we aimed to conduct human epidemiological studies to infer a causal relationship between GM and DL. In addition, because GM in women and men are inherently different (Kim et al., 2020), male and female groups were classified for analysis in this study. Thus, the objective of this study was to use the LiNGAM to infer a causal relationship between GM and blood lipid profiles in Japanese adults.

## Materials and methods

### Participants

The participants were 234 residents (109 men and 125 women) aged 40 years or older, of Shika-machi, Hakui-gun, Ishikawa Prefecture, Japan, whose fecal samples were collected during a health checkup in January 2020. The DL diagnosis was based on the following criteria: LDL-C ≥ 140 mg/dL, HDL-C < 40 mg/dL, and TG ≥ 150 mg/dL (Kinoshita et al., 2018). We excluded 1) individuals taking lipid-lowering drugs, antibiotics, steroids, bowel regulators, biocides, antibacterial agents, and proton pump inhibitors; 2) patients undergoing treatment for cancer, 3) individuals who had eaten within 10 h at the time of blood collection; and 4) individuals with missing data.

### Data source

Data from the Shika-machi Super Preventive Health Examination, a population survey aimed at establishing preventive methods for lifestyle-related diseases, were used. The survey was conducted between December 2019 and January 2020. The four model districts selected from the Shika area were Horimatsu, Higashimasuho, Tsuchida, and Higashiki (Karashima et al., 2018; Nagase et al., 2020).

### Ethical considerations

This study was approved by the Human Research Ethics Committee of Kanazawa University (approval number: 1491) and conducted in accordance with the principles of the Declaration of Helsinki and the Kanazawa University Microbial Safety Management Regulations. After providing an overview of the study to all participants at the time of physical examination, written informed consent prior to GM collection was obtained. The fecal samples were processed in a Biosafety Level 2 laboratory.

### Data collection

The Shika-machi Super Preventive Health Checkup data regarding parameters such as age, sex, medical history, medication status, and alcohol consumption/smoking status were collected using a questionnaire. The body mass index (BMI) was calculated by dividing the current weight (kg) by the square of the height (m^2^). After a 12-hour fasting, venous blood was collected to measure the glutamate-oxaloacetate transaminase (GOT), glutamate-pyruvate transaminase (GPT), gamma-glutamyl transferase (γ -GTP), alkaline phosphatase, amylase, hemoglobin, hemoglobin A1c, fasting glucose, insulin, serum creatinine (S-Cre), serum sodium, serum potassium, serum chloride (S-Cl), TG, total cholesterol (TC), LDL-C, and HDL-C levels. The daily salt intake was calculated based on the 24-hour urinary creatinine and sodium excretion values (Umemura et al., 2019). The estimated glomerular filtration rate was calculated using the serum creatinine levels.

### Fecal sample collection and DNA extraction

Fecal samples were collected from 234 participants using a method described previously with slight modifications (Nagase et al., 2020). The stool surface samples were collected independently by the participants using clean paper (AS ONE, Osaka, Japan) and a clean spatula with a plastic tube (AS ONE, Japan). The collected fecal samples were kept on ice and transported to the laboratory. The samples were stored at −80°C until DNA extraction. The total DNA extraction was performed using the NucleoSpin^®^ DNA Stool (Machery-Nagel, Dürren, Germany).

### Next-generation sequencing

The DNA extracted from the GM was processed for identification of the 16S rRNA gene sequence by NGS, using a previously described method (Nagase et al., 2020). The 16S rRNA gene was amplified using the 1st PCR primers (F: 5′-TCG TCG GCA GCG TCA GAT GTG TAT AAG AGA CAG CCT ACG GGN GGC WGC AG-3′; R: 5′-GTC TCG TGG GCT CGG AGA TGT GTA TAA GAG ACA GGA CTA CHV GGG TAT CTA ATC C -3′) (Kameoka et al., 2021) (Hokkaido system science Co., Ltd., Osaka, Japan). Ex Taq^®^ hot-start version of DNA polymerase and TaKaRa PCR Thermal Cycler Dice^®^ Gradient (TaKaRa Bio Inc., Shiga, Japan) were used to sequence the V3-V4 region of the 16S rRNA gene. Polymerase chain reaction (PCR) products were purified using Agencourt AMPure XP magnetic beads (Beckman Coulter, Inc., CA, USA). The concentrations of the resultant PCR products were measured using the Qubit^®^dsDNA HS Assay Kit and Qubit^®^ 3.0 Fluorometer (Thermo Fisher Scientific). All the purified PCR products were analyzed using MiSeq (Illumina, Inc., CA, USA) after processing.

### Microbiome analysis

For microbiome analysis, QIIME2 software was used (Qiime2 docs). Demultiplexed paired-end sequence data were denoised with DADA2, and the Silva 16S rRNA database (release 132) naïve Bayes classifier was used for ASV classification (Quast et al., 2013). Samples with fewer than 5000 sequences were removed from the analysis.

### Statistical analysis

Python (version 3.8.8) with the scikit-learn package (version 0.24.1) (Pedregosa et al., 2011) or R, using R-studio (version 4.1.1) (RStudio, Boston, MA, United States), was used for statistical analysis and machine learning.

The clinical information of the participants was tested for normality of distribution using the Shapiro-Wilk test. Normally distributed data were presented as mean ± standard deviation, and non-normally distributed data were presented as median (25^th^–75^th^ percentile). The differences in the clinical information between the groups were tested for significance using a one-way analysis of covariance (ANCOVA) for normally distributed data and ANCOVA with rank ordering (Quade’s non-parametric ANCOVA) (Quade, 1967) for non-normally distributed data. The relative proportions of the GM in each group were compared using Quade’s non-parametric ANCOVA and Tukey’s HSD test. Confounders that may have affected the GM [age, sex, BMI, daily salt intake, and weekly frequency of alcohol intake (Vujkovic-Cvijin et al., 2020)] were adjusted for and assessed. In addition, clinical background, which showed significant differences between the hyperlipidemic and non-hyperlipidemic groups, was added as a new confounding factor. The significance level of all the tests was set at *P* < 0.05. The beta diversity between each participant group was assessed using the non-metric multidimensional scaling analysis with the Bray–Curtis of R’s “package vegan” and the permutation multivariate analysis of variance (Dixon, 2003). Alpha diversity was assessed using the Chao1 index with commands within QIIME2 and 97% of observed. It was evaluated based on operational classification units. This value was evaluated at a sampling depth of 5000, and samples that did not meet 5000 were excluded (Willis, 2019).

The least absolute shrinkage and selection operator logistic regression model (LASSO logistic regression) was used to calculate the odds ratios and *P*-values using R’s “Package glmnet” (Tibshirani, 1996). A linear discriminant analysis effect size (LEfSe) was used to identify the GMs associated with DL (Segata et al., 2011). Correlation coefficients and *P*-values were calculated using Spearman’s rank correlation coefficient in R’s “Package ppcor” after adjusting for the variables listed above. The correlation coefficients were plotted using “Package pheatmap.” The direct LiNGAM was built using “LiNGAM” in Python (Shimizu et al., 2011; Kotoku et al., 2020). Bacterial genera that were significantly associated with dyslipidemia or the four lipid profiles in ANCOVA, LEfSe, LASSO logistic regression, or correlation analysis were used for LiNGAM.

## Results

### Clinical background

Of the 234 participants who submitted fecal samples, four were excluded because they had less than 5000 sequences in the NGS analysis. Fifty-seven were taking lipid-lowering drugs, antibiotics, steroids, bowel regulators, biocides, antibacterials, and proton pump inhibitors; two were undergoing cancer treatment; and 10 were not fasting at the time of blood collection. Finally, a total of 161 participants (79 men and 82 women) were analyzed.

### Differences in GM composition by sex

**Figure 1:**
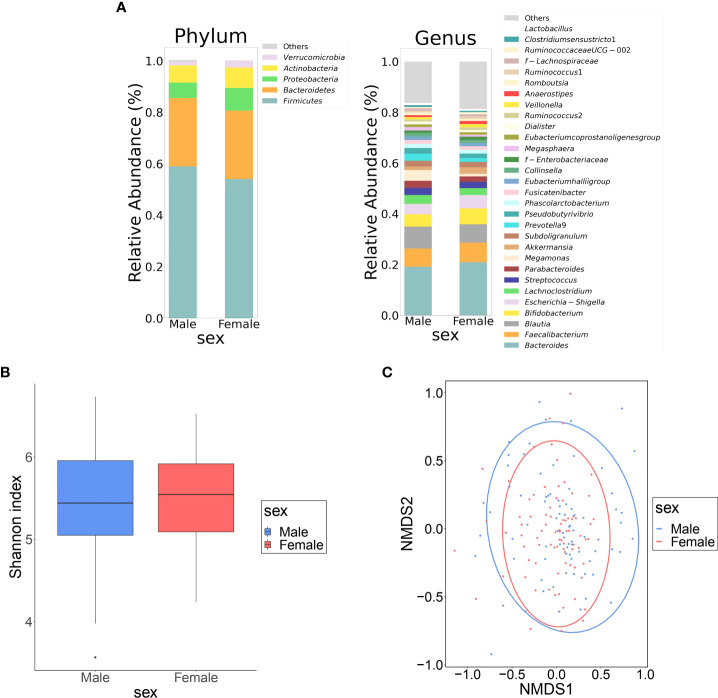
Differences in gut microbiota between female and male groups. **(A)** Comparison of relative abundance ratios at the phylum and genus level for the top 30 bacterial genera with mean abundance ratios by sex. **(B)** The difference in α-diversity calculated using Shannon index, 5,000 depths. One sample that failed to be sequenced was excluded. (*P* = 0.490, Quade’s nonparametric ANCOVA) **(C)** Plot of β-diversity analysis calculated by NMDS ordering based on Bray-Curtis distance matrix. Red: female, blue: male. Ellipses represent 95% confidence intervals for each genus used in the analysis. (*P* = 0.953, PERMANOVA) NMDS: non-metric multidimensional scaling; ANCOVA: analysis of covariance; DL: dyslipidemia; PERMANOVA: permutation multivariate analysis of variance.

**Table 1** shows the clinical information of the male and female groups. There were significant differences in BMI, waist circumference, TC, TG, HDL-C, hemoglobin, fasting glucose, S-Cre, S-Cl, GOT, GPT, γ GTP, and frequency of alcohol consumption between the two groups. The composition of the top 30 genera of intestinal bacteria in the two groups at the level of phylum and genus is shown in **Figure 1A**. Non-metric multidimensional scaling analysis using genus diversity by Chao1 (α-diversity) and Bray-Curtis distance (β-diversity) showed no significant differences in gut microbial composition between the two groups (**Figures 1B, C**).

**Table 1:** Characteristics of study participants categorized by sex.

	Male	Female	*P*-value
	79	82	
Age (years)	66 (55-70)	61 (55-67)	0.243
BMI (kg/m2)	24.2 ± 3.1	22.4 ± 3.3	< 0.001
Waist circumference (cm)	86.0 (82.3-91.5)	82.0 (74.9-87.4)	< 0.001
TC (mg/dL)	210 (189-235)	226 (205-260)	< 0.001
TG (mg/dL)	98 (78-146)	80 (63-108)	0.002
LDL-C (mg/dL)	128 (108-142)	134 (116-156)	0.054
HDL-C (mg/dL)	60 (52-69)	75 (63-86)	< 0.001
SBP (mmHg)	135 ± 17	133 ± 18	0.487
DBP (mmHg)	80 ± 12	77 ± 11	0.139
Hemoglobin (g/dL)	15.4 (14.5-16.1)	13.6 (12.9-14.2)	< 0.001
Fasting glucose (mg/dL)	99 (94-111)	93 (88-98)	< 0.001
HbA1c [NGSP] (%)	5.9 (5.6-6.1)	5.7 (5.5-5.9)	0.071
Insulin (μU/mL)	4.53 (3.33-7.32)	3.29 (3.29-6.78)	0.712
S-Cre (mg/dL)	0.89 (0.82-1.02)	0.66 (0.61-0.73)	< 0.001
eGFR (mL/min/1.73m^2^)	66 (59-75)	69 (62-77)	0.264
S-Na (mEq/L)	142 (141-143)	142 (141-144)	0.294
S-K (mEq/L)	4.2 (4.1-4.6)	4.2 (3.9-4.4)	0.087
S-Cl (mEq/L)	103 (102-105)	104 (103-106)	0.005
GOT (IU/L)	25 (22-29)	23 (20-26)	0.005
GPT (IU/L)	22 (16-31)	16 (14-20)	< 0.001
γ-GTP (IU/L)	35.0 (23.0-55.5)	18.0 (15.0-30.8)	< 0.001
ALP (IU/L)	212.0 (185.5-244.5)	225.0 (192.0-269.0)	0.082
Amylase (IU/L)	85.0 (68-102)	82 (65-100)	0.676
Alcohol consumption (day/week)	6.0 (0-7)	0 (0-2)	< 0.001
Smoking (cigarettes/day)	0 (0-0)	0 (0-0)	0.060
Daily salt intake (g/day)	9.8 ± 2.5	9.7 ± 2.0	0.627

The P-values were calculated by covariance analysis (ANCOVA or Quade’s non-parametric ANCOVA). Abbreviations: ANCOVA, analysis by covariance; BMI, body mass index; TC, total cholesterol; TG, triglycerides; LDL-C, low-density lipoprotein cholesterol; HDL-C, high-density lipoprotein cholesterol; SBP, systolic blood pressure; DBP, diastolic blood pressure; HbA1c, hemoglobin A1c; NGSP, National Glycohemoglobin Standardization Program; S-Cre, Serum creatinine; eGFR, estimated glomerular filtration rate; S-Na, Serum sodium; S-K, Serum potassium; S-Cl, serum chloride; GOT, glutamate-oxaloacetate transaminase; GPT, glutamate-pyruvate transaminase; γ -GTP, gamma-glutamyl transferase; ALP, alkaline-phosphatase

### Differences in the GM composition with and without DL

**Figure 2:**
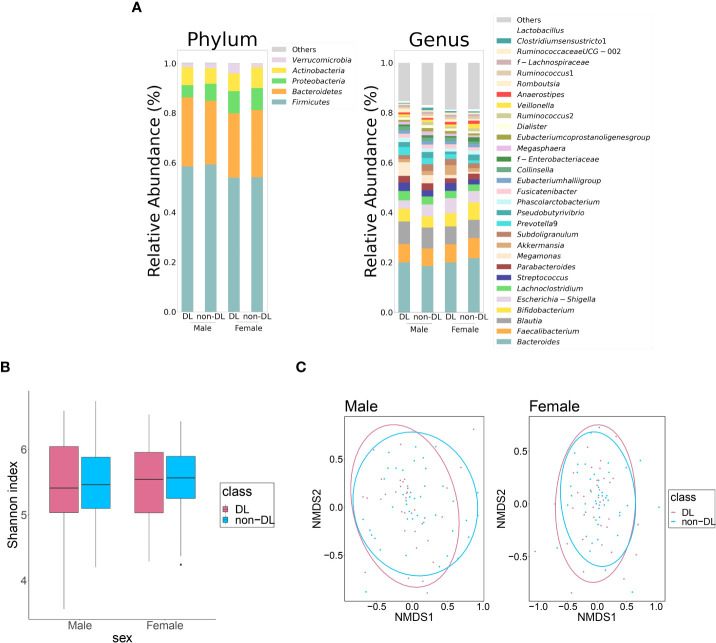
Differences in the gut microbiota between DL and non-DL groups. **(A)** Comparison of relative abundance ratios at the phylum and genus level for the top 30 bacterial genera with mean abundance ratios according to the presence or absence of DL. **(B)** Differences in the α-diversity calculated using Shannon index, 5,000 depths. One sample that failed to be sequenced were excluded. (Men: *P* = 0.922, Women: *P* = 0.559, Quade’s non-parametric ANCOVA) **(C)** Plot of the β-diversity analysis calculated by NMDS ordination based on Bray-Curtis distance matrix. Red: DL group; blue: non-DL group. Ellipses represent the 95% confidence interval for each genus in the analysis. (Men: *P* = 0.422, Women: *P* = 0.407, PERMANOVA).

**Table 2** shows the clinical information of DL and non-DL groups among the male and female participants. The prevalence of DL was 43.0% and 43.9% in men and women, respectively. There were significant differences in TC and LDL-C between the DL and non-DL groups for both men and women. In addition, there was a significant difference in the TG levels between the DL and non-DL groups in men. The composition of the top 30 genera of intestinal bacteria in the DL and non-DL groups is shown in **Figure 2A** at the level of phylum and genus. There was no significant difference in GM composition between DL and non-DL groups in terms of genus α- and β-diversity in both sexes (**Figures 2B, C**).

**Table 2:** Characteristics of the study participants classified based on the presence or absence of dyslipidemia.

	Male		Female	
Characteristic	Non-DL group	DL group	Non-DL group	DL group
n	n=45	n=34	n=46	n=36
Age (years)	66 (59-70)	64 (49-67)	61 (55-67)	61 (55-70)
BMI (kg/m2)	23.4 ± 2.8	25.0 ± 3.1 *	22.0 ± 3.1	23.0 ± 2.5
Waist circumference (cm)	84.3 (7.90-89.9)	90.0 (84.1-95.4) *	80.6 (73.3-86.8)	83.5 (75.6-88.0)
TC (mg/dL)	198 (182-210)	234 (219-255) *	211 (196-223)	264 (254-273) *
TG (mg/dL)	83 (67-101)	151 (108-201) *	78 (62-96)	80 (64-132)
LDL-C (mg/dL)	115.0 (100.0-129.0)	144.0 (125.5-164.8) *	121.5 (106.2-131.8)	158.5 (147.0-172.2) *
HDL-C (mg/dL)	60 (54-72)	59 (45-68)	74 (64-86)	75 (60-84)
SBP (mmHg)	133 ( ± 17)	138 ± 17	131 ± 18	135 ± 3
DBP (mmHg)	78 ± 11	82 ± 12	76 ± 11	78 ± 10
Hemoglobin (g/dL)	14.90 (13.80-15.70)	15.90 (15.25-16.40) *	13.90 (13.18-14.22)	13.55 (12.90-14.18)
Fasting glucose (mg/dL)	98.0 (93.0-111.0)	103.9 (95.0-110.8)	92.5 (89.0-98.0)	92.5 (86.0-98.5)
HbA1c [NGSP] (%)	5.8 (5.5-6.3)	5.9 (5.6-6.0)	5.7 (5.5-5.9)	5.8 (5.6-6.0)
Insulin (μU/mL)	4.22 (2.51-6.22)	6.09 (4.27-8.44) *	4.90 (3.51-6.38)	4.28 (3.04-7.40)
S-Cre (mg/dL)	0.89 (0.81-1.00)	0.92 (0.83-1.04)	0.66 (0.60-0.71)	0.69 (0.64-0.76)
eGFR (mL/min/1.73m^2^)	69 (59-75)	65 (58-74)	70 (65-78)	67 (61-73)
S-Na (mEq/L)	142 (141-143)	142.5 (141-143)	142.0 (141-144)	143 (142-144)
S-K (mEq/L)	4.3 (4.1-4.6)	4.2 (4.1-4.4)	4.2 (4.0-4.4)	4.2 (3.9-4.4)
S-Cl (mEq/L)	103 (102-105)	103 (102-104)	104 (103-106)	104 (103-105)
GOT (IU/L)	25 (22-29)	26 (21-30)	22 (19-26)	23 (21-26)
GPT (IU/L)	18 (15-26)	28 (21-35) *	15 (12-18)	19 (14-24) *
γ-GTP (IU/L)	29.0 (20.0-43.0)	41.0 (30.0-61.3) *	16.0 (14.0-25.3)	22.5 (16.8-33.3) *
ALP (IU/L)	213.0 (187.0-253.0)	205.5 (182.2-239)	227.0 (192.8-268.0)	217.0(19.2-277.8)
Amylase (IU/L)	89 (70-102)	78 (65-100)	83 (73-99)	79 (61-105)
Frequency of alcohol consumption (day/week)	4 (0-7)	6 (1-7)	0 (0-2)	0 (0-2)
Smoking (cigarettes/day)	0 (0-15)	0 (0-0)	0 (0-0)	0 (0-1.25)
Daily salt intake (g/day)	10.0 ± 2.4	9.6 ± 2.6	9.8 ± 2.1	9.4 ± 1.8

*: P < 0.05, vs Non-DL group; analysis by covariance (ANCOVA or Quade’s non-parametric ANCOVA). Abbreviations: ANCOVA, analysis by covariance; BMI, body mass index; TC, total cholesterol; TG, triglycerides; LDL-C, low-density lipoprotein cholesterol; HDL-C, high-density lipoprotein cholesterol; SBP, systolic blood pressure; DBP, diastolic blood pressure; HbA1c, hemoglobin A1c; NGSP, National Glycohemoglobin Standardization Program; S-Cre, Serum creatinine; eGFR, estimated glomerular filtration rate; S-Na, Serum sodium; S-K, Serum potassium; S-Cl, serum chloride; GOT, glutamate-oxaloacetate transaminase; GPT, glutamate-pyruvate transaminase; γ -GTP, gamma-glutamyl transferase; ALP, alkaline-phosphatase

### Comparison of GM between the DL and non-DL groups

**Figure 3:**
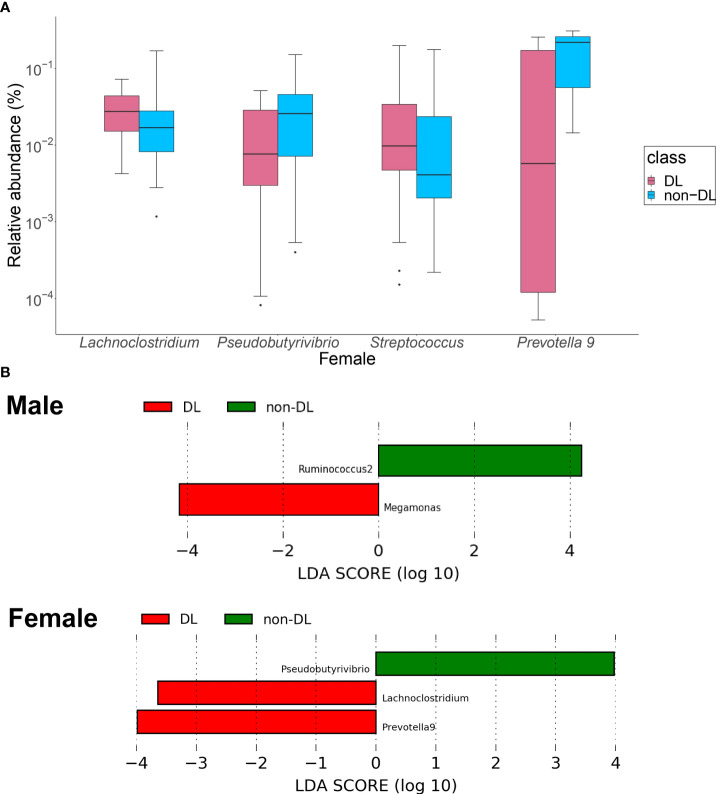
Identification of the intestinal bacteria involved in DL. **(A)** Bacterial genera with significantly different relative to the abundance ratios in the presence and absence of DL. Red: DL group, blue: non-DL group. **(B)** LEfSe analysis of the top 30 bacterial species, with LDA score = 2.0 as the cutoff value. DL: dyslipidemia; LEfSe: linear discriminant analysis effect size.

Among the top 30 bacterial genera, the relative amounts of four genera, in terms of the percentage of the GM present in the gut of the participants, were significantly different between the DL and non-DL groups in women (**Figure 3A**): the relative amount of *Lachnoclostridium* and *Streptococcus* were significantly higher (*P* = 0.003, 0.018), the relative amount of *Pseudobutyrivrio* and *Prevotella 9* were lower in the DL group (*P* = 0.032, 0.041). There was no significant difference of bacterial genus in men.

In the LEfSe analysis of the top 30 bacteria (**Figure 3B**), *Megamonas* showed a significantly higher presence ratio in the DL group in men (linear discriminant analysis score = 4.16, *P* = 0.021), and *Ruminococcus2* had a significantly higher presence ratio in the non-DL group (linear discriminant analysis score = 4.25, *P* = 0.024). In women, *Lachnoclostridium* and *Prevotella 9* had significantly higher presence ratio in the DL group (linear discriminant analysis score = 3.56, 3.95, *P* =0.013, 0.029, respectively). *Psudobutyrivibrio* had significantly higher presence ratio in the non-DL group (linear discriminant analysis score = 3. 95, *P* =0.040).

### Selection of GMs for the DL and non-DL classification

The LASSO logistic regression model was used to select the GMs that contribute to the prediction of DL and non-DL classification. In men, a model was developed with the area under the ROC curve (AUC) 0.540, sensitivity 0.735, and specificity 0.422, but no characteristic bacterial genus was identified. In women, the model with AUC 0.819, sensitivity 0.750, and specificity 0.848 was developed (**Supplementary Figure 1**). The odds ratios suggested that *Akkermansia* and *Lachnoclostridium* may be associated with DL (**Table 3**). Variables that were not significantly different but were selected are shown in **Supplementary Table 1**.

**Table 3:** Bacteria selected by the least absolute shrinkage and selection operator logistic model.

Sex	Bacteria	Odds ratio	Lower 95% CI	Upper 95% CI	*P-*value
Female	*Akkermansia*	1.149	1.016	1.299	0.0269
	*Lachnoclostridium*	1.439	1.065	1.944	0.0178

Analysis by LASSO logistic model. A LASSO logistic model was constructed to identify variables predicting dyslipidemia. Among the top 30 bacteriological features with mean proportion present, no bacterial genera were selected in men. In women, Akkermansia and Lachnoclostridium were selected. All of these selected factors did not show multicollinearity in a model with a variance inflation factor <10.

CI, confidence interval; E/S, Escherichia/Shigella.

### Correlation and causal diagram between lipids and GM

**Figure 4:**
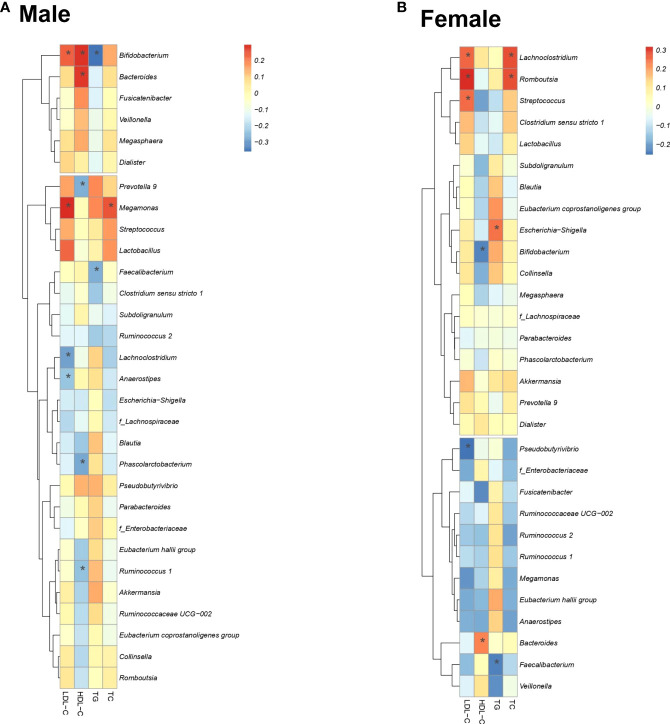
Correlation of four lipid profiles with important bacteria. Correlation between the lipid profiles and the presence ratio of three important bacteria. Spearman’s correlation coefficient determines the color intensity of the heat map. Red: positive correlation, blue: negative correlation. (*: *P* < 0.05).

**Figure 4** shows the correlation between lipid profiles and the relative abundance of the three selected gut bacteria for each sex. In men, there was a significant correlation between nine bacterial genera (*Bifidobacterium, Bacteroides, Megamonas, Prevotella 9, Phascolarctobacterium*, *Lachnoclostridium, Faecalibacterium, Anaerostipes*, *Ruminococcus 1*) and four lipid profiles (**Figure 4A**). In women, there was a significant positive correlation between eight bacterial genera (*Pseudobutyrivibrio, Streptococcus, Lachnoclostridium, Romboutsia, Escherichia/Shigella* (*E/S*)*, Bacteroides, Bifidobacterium, Faecalibacterium*) and four lipid profiles (**Figure 4B**).

**Figure 5:**
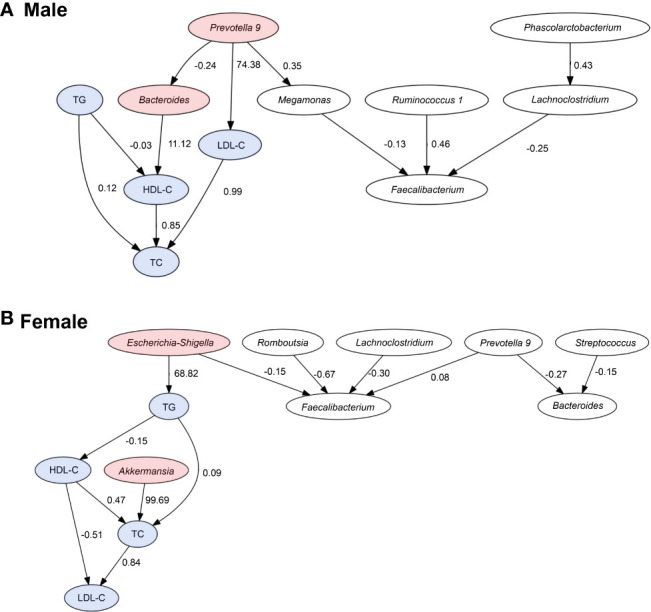
Causal relationships between the lipid profiles and important bacteria. Arrows indicate the causal relationship between the two connected indicators. Indicators with no causal relationship between them were not shown in the figure. Red: bacteria with an estimated causal relationship with lipids, blue: lipid profile indicators. The values are absolute values of partial regression coefficients.

The LiNGAM algorithm was used to infer the causal relationship between the lipid profiles and the presence ratio of the gut bacteria. The inferred causal relationship diagrams, causal order, and partial regression coefficient of the four lipid profiles for the three selected gut bacteria for each sex are shown in **Figure 5**. The arrows represent the estimated causal relationship between the two linkage indices with non-zero partial regression coefficients.

In men, the estimated causal relationships were seen in *Prevotella 9* with a positive effect on LDL-C levels (coefficient = 74.38), *Bacteroides* with a positive effect on HDL-C levels (coefficient = 11.12). In women, genus *Akkermansia* had a positive effect on TC levels (coefficient = 99.69) and genus *E/S* had a positive effect on TG levels (coefficient = 68.82).

## Discussion

The lipid profile of Japanese adults may be associated with changes in the abundance ratios of certain gut bacteria. In this study, direct LiNGAM estimated the causal relationship between different bacteria and lipid profiles in men and women. Some reports have suggested that biological sex difference and sex hormones themselves cause changes in GM (Shin et al., 2019; Kim et al., 2020). It is also possible that differences in clinical background parameters due to gender may have affected the composition of GM. In the present study, the frequency of alcohol consumption and levels of hepatobiliary enzymes were significantly higher in men than in women. Alcohol consumption and alcoholic liver disease have been reported to be closely related to GM changes (Zhong and Zhou, 2014; Vujkovic-Cvijin et al., 2020). In summary, sex difference, lifestyle, and clinical backgrounds of men and women may be responsible for the differences in gut bacteria associated with DL.

In men, LiNGAM showed that *Bacteroides* and *Prevotella 9* were shown to be potentially related to changes in HDL-C or LDL-C levels. In the lipid metabolic pathway, cholesteryl ester transfer protein (CETP) plays a major role in the synthesis of HDL-C. CETP is a plasma glycoprotein synthesized in the liver and small intestine that facilitates the transfer of cholesteryl esters from HDL-C to lipoproteins, such as apolipoprotein B. High CETP activity decreases the concentration of circulating HDL-C (Tall, 1993). The mechanism of GM-induced changes in blood HDL-C levels may involve CETP activity and lipopolysaccharide (LPS). LPS is a molecule composed of lipids and carbohydrates, found on the cell wall surface of gram-negative bacteria. LPS is activated in liver Kupffer cells (KCs), which in turn leads to decreased CETP activity in the plasma and increases HDL (Hartstra et al., 2014; van der Tuin et al., 2018). Yoshida et al. reported that oral administration of *Bacteroides* in mice prone to atherosclerosis reduced lipopolysaccharide production by intestinal microorganisms and suppressed the formation of atherosclerotic lesions (Yoshida et al., 2018). The genus *Bacteroides* is also abundant in the intestines of healthy adults (The human microbiome project consortium, 2012), and many studies have reported that they have anti-obesity effect (Xu et al., 2022). Yoshida et al. reported *Bacteroides* spp. promoted branched-chain amino acid catabolism in brown fat in mice (Yoshida et al., 2021).

The LinGAM results suggest that *Prevotella* plays multiple roles, including increasing LDL-C alone and decreasing HDL-C by forming a network with *Bacteroides*. The possibility that intestinal bacteria form a network and affect lipids is a very interesting result. In a previous study, *Prevotella copri*, the most abundant species of the genus *Prevotella*, was reported to cause weight loss and lower cholesterol levels (Tett et al., 2021). However, in the present population, *Prevotella* was inferred to increase the risk of DL. Whether the effect of *Prevotella* on lipids is due to population ratio, dietary habits, or the effect of interactions between intestinal bacteria is unknown and requires further study.

In women, LiNGAM results inferred a causal relationship between *Akkermansia* and *E/S* with lipid profiles. *A. muciniphila*, one of the genus *Akkermansia*, has been reported to affect lipid metabolism in various studies (Dao et al., 2016; Xu et al., 2020).

*Escherichia coli* and *Shigella* spp. are closely related since both bacteria belong to the family *Enterobacteriaceae*. Phenotypically, although *E. coli* and *Shigella* spp. share many similarities, they are considered separate entities epidemiologically and clinically. However, the 16S rRNA method cannot accurately distinguish these two bacteria. *E/S* has been reported to be associated with lifestyle-related diseases, such as DL and Non-alcoholic fatty liver disease (NAFLD). Cohort studies have reported that the *E/S* ratio is significantly higher in obese individuals (Maskarinec et al., 2021; Jin et al., 2021). The genus *Escherichia* is increased in patients with NAFLD (Fukui, 2019). Furthermore, *Escherichia fergusonii* inhibits host lipid metabolism by inhibiting hepatic lipid β-oxidation and promoting *de novo* lipogenesis in non-obese rats (Xin et al., 2021).

This study has several limitations. First, the study involves a small-sized cohort; nevertheless, the unification of region and sex in this study may have made a clearer association between GM and disease. Second, we did not examine the bacteria at the species level since that would require a whole-genome analysis and not just the V3–V4 region of the GM rRNA. Finally, the present results are inferences of cause and effect based on observational study data, and further investigations, such as metabolomic analysis using animal models, are necessary to clarify the mechanism and the effects of the changes in the abundance of specific intestinal bacteria on lipids.

In conclusion, we identified *Bacteroides* and *Prevotella 9* in men, and *E/S* and *Akkermansia* in women, to be potentially causally related GM with serum lipid profile levels. This suggests that maintaining a high ratio of specific intestinal bacteria may lead to the maintenance of lipid profiles, and eventually reducing the risk of atherosclerosis. In the future, it may be possible to identify a GM composition that improves lipid metabolism and contributes to the prevention of CVD; sex-based lifestyle changes that target GM can also be explored accordingly. Furthermore, the administration of probiotics containing *Bacteroides* in men may be a new therapeutic strategy for patients with DL. Further studies are required to elucidate the detailed mechanisms of GM in DL.

## Data availability statement

The data presented in the study are deposited in the DDBJ repository, accession number DRA013759.

## Ethics statement

The studies involving human participants were reviewed and approved by the Kanazawa University Hospital Human Research Ethics Committee. The patients/participants provided their written informed consent to participate in this study.

## Author contributions

SK and SO designed the study and evaluated and edited the manuscript. KaO, KoO, MiK, DA, MD, TY, HT, AH, and HidN supervised the consultations. YM, KT, and SO collected the samples and performed the experiments. YM, SK, and KaO performed the statistical analyses. YM, SK, KaO, MaK, and HirN contributed to the analysis and interpretation of the data. YM and SK wrote the manuscript. SK and SO acquired funding for the research and supervised the study. All authors have checked and approved the final version of the manuscript.

## Funding

This study was supported by a grant from JSPS KAKENHI [grant numbers JP19K17956 and JP21K10392 to SK] and Yakult Bio-Science Foundation. The funder financed the study experiments as well as the writing and proofreading of this manuscript.

## Acknowledgments

We thank Editage (Tokyo, Japan; www.editage.jp) for the English language editing. We also thank Dr. Satoshi Nagase, Dr. Miki Matsue, Ms. Ayaka Matsuoka, Ms. Haruka Ishihara, Ms. Akane Yonezawa, Ms. Yurina Yamatsu, and Mr. Reo Fukuda (Department of Clinical Laboratory Science, Faculty of Health Sciences, Institute of Medical, Pharmaceutical and Health Sciences, Kanazawa University, Kanazawa, Japan) for assisting with the sample collection. We thank Dr. Takayuki Kuraishi (Laboratory of Host Defense and Responses, Faculty of Pharmacy, Institute of Medical, Pharmaceutical and Health Sciences, Kanazawa University, Kanazawa, Japan) for running the analysis using Miseq.

## Conflict of interest

The authors declare that the research was conducted in the absence of any commercial or financial relationships that could be construed as a potential conflict of interest.

## Publisher’s note

All claims expressed in this article are solely those of the authors and do not necessarily represent those of their affiliated organizations, or those of the publisher, the editors and the reviewers. Any product that may be evaluated in this article, or claim that may be made by its manufacturer, is not guaranteed or endorsed by the publisher.

## Abbreviations

DL, dyslipidemia; CVD, cardiovascular disease; LDL-C, low-density lipoprotein cholesterol; HDL-C, high-density lipoprotein cholesterol; TG, triglycerides; GM, gut microbiota; LiNGAM, linear non-Gaussian acyclic model; BMI, body mass index; TC, total cholesterol; PCR, polymerase chain reaction; ANCOVA, analysis of covariance; LASSO, least absolute shrinkage and selection operator; LEfSe, linear discriminant analysis effect size; NAFLD, non-alcoholic fatty liver disease; CETP, cholesteryl ester transfer protein; LPS, lipopolysaccharide; *E/S*, *Escherichia/Shigella*.
