# Supplementary material for: Impact of gut microbiome on dyslipidemia in japanese adults: Assessment of the Shika-machi super preventive health examination results for causal inference
Source: Front Cell Infect Microbiol. 2022 Sep 2;12:908997. doi: 10.3389/fcimb.2022.908997 (PMC9479221; doi:10.3389/fcimb.2022.908997)
Supplement: Supplementary file 1 [file Table_1.docx]

Supplementary Material

Supplementary Tables

**Supplementary Table 1.**

All features selected by the least absolute shrinkage and selection operator logistic model (related to Table 3).

| Charastaristic | Odds ratio | lower.95ci | upper.95ci | *P*-value |
| --- | --- | --- | --- | --- |
| Male |  |  |  |  |
| *Eubacterium hallii.group* | 0.931 | 0.705 | 1.229 | 0.613 |
| *Akkermansia* | 0.976 | 0.858 | 1.110 | 0.708 |
| *Anaerostipes* | 1.057 | 0.662 | 1.686 | 0.817 |
| *Dialister* | 0.953 | 0.756 | 1.201 | 0.682 |
| *Megasphaera* | 0.964 | 0.811 | 1.146 | 0.679 |
| Female |  |  |  |  |
| *Eubacterium*  *coprostanoligenes.group* | 1.237 | 0.861 | 1.779 | 0.250 |
| *Eubacterium hallii group* | 0.960 | 0.452 | 2.039 | 0.915 |
| *Escherichia-Shigella* | 1.021 | 0.952 | 1.094 | 0.564 |
| *Bifidobacterium* | 0.949 | 0.859 | 1.049 | 0.309 |
| *Blautia* | 0.939 | 0.804 | 1.098 | 0.433 |
| *Clostridium.sensu.stricto.1* | 0.976 | 0.613 | 1.554 | 0.919 |
| *Collinsella* | 1.587 | 0.927 | 2.716 | 0.092 |
| *Dialister* | 1.047 | 0.654 | 1.675 | 0.848 |
| *Megamonas* | 1.065 | 0.932 | 1.218 | 0.355 |
| *Parabacteroides* | 0.794 | 0.557 | 1.132 | 0.203 |
| *Phascolarctobacterium* | 0.737 | 0.518 | 1.048 | 0.090 |
| *Prevotella.9* | 1.021 | 0.926 | 1.125 | 0.674 |
| *Ruminococcus.1* | 1.398 | 0.864 | 2.264 | 0.173 |
| *Ruminococcus.2* | 0.666 | 0.417 | 1.064 | 0.089 |
| *Streptococcus* | 1.184 | 0.987 | 1.419 | 0.069 |
| *Veillonella* | 0.758 | 0.541 | 1.063 | 0.109 |

**Supplementary Figure 1.**

ROC curves for the female The LASSO logistic regression model.


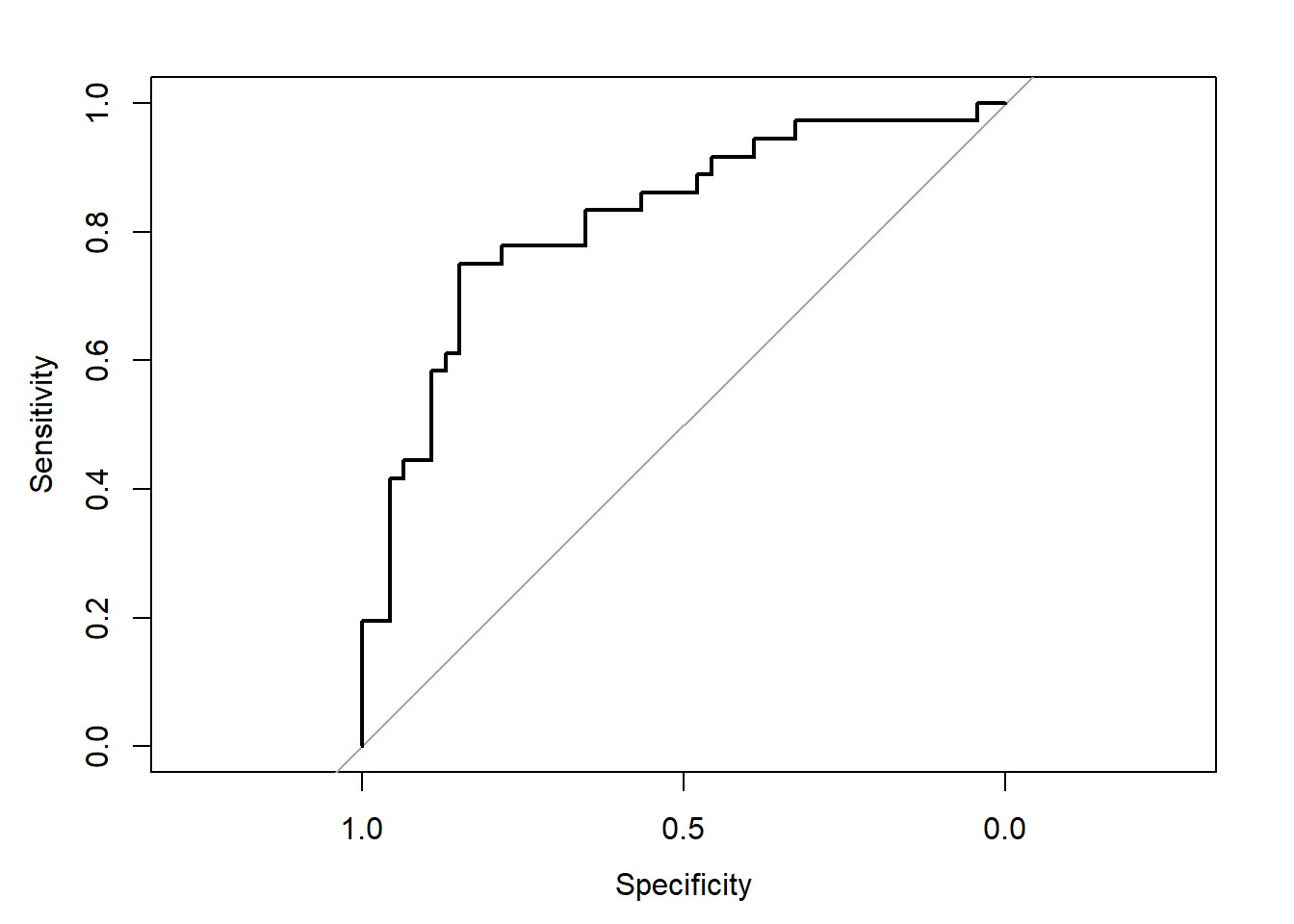


**Supplementary Table 2.**

List of Bio sample IDs used for the analysis from the raw sequencing registered in the DNA Data Bank of Japan (DDBJ) (Numbers DRA13759).

| Male | | Female | |
| --- | --- | --- | --- |
| DL | non-DL | DL | non-DL |
| SAMD00445753 | SAMD00445756 | SAMD00445755 | SAMD00445752 |
| SAMD00445754 | SAMD00445758 | SAMD00445757 | SAMD00445763 |
| SAMD00445760 | SAMD00445767 | SAMD00445762 | SAMD00445764 |
| SAMD00445761 | SAMD00445769 | SAMD00445770 | SAMD00445765 |
| SAMD00445766 | SAMD00445776 | SAMD00445774 | SAMD00445772 |
| SAMD00445768 | SAMD00445779 | SAMD00445777 | SAMD00445778 |
| SAMD00445771 | SAMD00445783 | SAMD00445781 | SAMD00445780 |
| SAMD00445773 | SAMD00445785 | SAMD00445782 | SAMD00445784 |
| SAMD00445775 | SAMD00445786 | SAMD00445790 | SAMD00445787 |
| SAMD00445794 | SAMD00445788 | SAMD00445792 | SAMD00445791 |
| SAMD00445799 | SAMD00445797 | SAMD00445796 | SAMD00445795 |
| SAMD00445800 | SAMD00445806 | SAMD00445798 | SAMD00445802 |
| SAMD00445820 | SAMD00445810 | SAMD00445801 | SAMD00445805 |
| SAMD00445828 | SAMD00445816 | SAMD00445803 | SAMD00445817 |
| SAMD00445829 | SAMD00445818 | SAMD00445804 | SAMD00445822 |
| SAMD00445831 | SAMD00445819 | SAMD00445807 | SAMD00445837 |
| SAMD00445834 | SAMD00445821 | SAMD00445808 | SAMD00445838 |
| SAMD00445835 | SAMD00445824 | SAMD00445809 | SAMD00445842 |
| SAMD00445847 | SAMD00445825 | SAMD00445812 | SAMD00445843 |
| SAMD00445854 | SAMD00445826 | SAMD00445823 | SAMD00445845 |
| SAMD00445855 | SAMD00445830 | SAMD00445827 | SAMD00445848 |
| SAMD00445858 | SAMD00445832 | SAMD00445836 | SAMD00445849 |
| SAMD00445868 | SAMD00445833 | SAMD00445844 | SAMD00445852 |
| SAMD00445874 | SAMD00445839 | SAMD00445862 | SAMD00445853 |
| SAMD00445879 | SAMD00445840 | SAMD00445867 | SAMD00445856 |
| SAMD00445890 | SAMD00445841 | SAMD00445883 | SAMD00445857 |
| SAMD00445893 | SAMD00445846 | SAMD00445888 | SAMD00445860 |
| SAMD00445897 | SAMD00445850 | SAMD00445896 | SAMD00445864 |
| SAMD00445900 | SAMD00445861 | SAMD00445904 | SAMD00445866 |
| SAMD00445902 | SAMD00445863 | SAMD00445906 | SAMD00445869 |
| SAMD00445903 | SAMD00445873 | SAMD00445909 | SAMD00445870 |
| SAMD00445908 | SAMD00445876 | SAMD00445916 | SAMD00445871 |
| SAMD00445918 | SAMD00445878 | SAMD00445921 | SAMD00445872 |
| SAMD00445919 | SAMD00445882 | SAMD00445923 | SAMD00445875 |
|  | SAMD00445884 | SAMD00518145 | SAMD00445877 |
|  | SAMD00445885 | SAMD00518146 | SAMD00445881 |
|  | SAMD00445889 |  | SAMD00445886 |
|  | SAMD00445892 |  | SAMD00445891 |
|  | SAMD00445894 |  | SAMD00445895 |
|  | SAMD00445905 |  | SAMD00445898 |
|  | SAMD00445910 |  | SAMD00445901 |
|  | SAMD00445911 |  | SAMD00445907 |
|  | SAMD00445914 |  | SAMD00445912 |
|  | SAMD00445917 |  | SAMD00445913 |
|  | SAMD00445922 |  | SAMD00445915 |
|  |  |  | SAMD00445920 |
